# G-quadruplexes are specifically recognized and distinguished by selected designed ankyrin repeat proteins

**DOI:** 10.1093/nar/gku571

**Published:** 2014-07-22

**Authors:** Oliver Scholz, Simon Hansen, Andreas Plückthun

**Affiliations:** Department of Biochemistry, University of Zurich, 8057 Zurich, Switzerland

## Abstract

We introduce designed ankyrin repeat binding proteins (DARPins) as a novel class of highly specific and structure-selective DNA-binding proteins, which can be functionally expressed within all cells. Human telomere quadruplex was used as target to select specific binders with ribosome display. The selected DARPins discriminate the human telomere quadruplex against the telomeric duplex and other quadruplexes. Affinities of the selected binders range from 3 to 100 nM. CD studies confirm that the quadruplex fold is maintained upon binding. The DARPins show different specificity profiles: some discriminate human telomere quadruplexes from other quadruplex-forming sequences like *ILPR, c-MYC and c-KIT*, while others recognize two of the sequences tested or even all quadruplexes. None of them recognizes dsDNA. Quadruplex-binding DARPins constitute valuable tools for specific detection at very small scales and for the *in vivo* investigation of quadruplex DNA.

## INTRODUCTION

The DNA double helix undoubtedly is one of the most important macromolecular structures. It folds independently of its sequence, provided two complementary DNA strands are available. This seeming generality made it a dogma that all cellular DNA exists in this conformation. However, depending on its sequence, DNA can also adopt different conformations, such as triple helices and quadruplexes, where three or four strands come together to form a common helix. In this article, we will focus on the investigation of quadruplex DNA. G-quadruplex DNA (G4) consists of stacks of planar G-quartets, where the four strands are connected via Hoogsteen base pairing and each guanine is donor and acceptor for two H-bonds (1,2). Different arrangements of G-quartets are possible, which differ in the orientation of the four DNA strands and their thermodynamic stability (3).

The relevance of DNA quadruplexes in biological systems is currently under intense discussion (1,4). Bioinformatics analyses predict 375,000 DNA sequences with the potential to form a quadruplex in the human genome (possibly quadruplex-forming sequences, PQS) (5). Their high frequency and non-random distribution make it possible that these sequence stretches exert important biological functions, even though direct evidence that this really occurs via quadruplex formation is sparse. PQS are not randomly distributed, but accumulate at promoter regions and in 5′ UTR, while the coding regions are depleted of PQS. The 5′ UTR quadruplexes have been proposed to most likely form and act on the RNA level (6). Formation of DNA quadruplexes is promoted whenever the complementary strands are separated, i.e. a situation occurring during transcription and DNA replication. Some PQS occur in repetitive sequences, like the insulin-linked polymorphic region (*ILPR*) (7,8) and the telomeric sequence.

**Figure 1.**
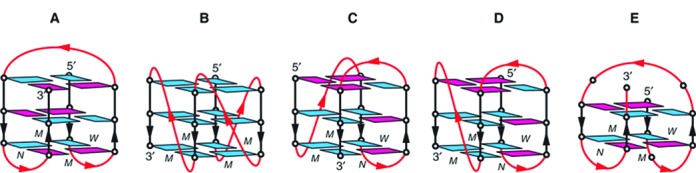
Schematic structure of human telomeric G-quadruplexes. (**A**) Basket-type form observed for d[A(GGGTTA)_3_GGG] in Na^+^ solution (53). (**B**) Propeller-type form observed for d[A(GGGTTA)_3_GGG] in a K^+^-containing crystal (54) (**C**) (3 + 1) "form 1" observed for d[TA(GGGTTA)_3_GGG] (55) and d[TTA(GGGTTA)_3_GGG] (10) in K^+^ solution. (**D**) (3 + 1) "form 2" observed for d[TA(GGGTTA)_3_GGGTT] (55) and d[TTA(GGGTTA)_3_GGGTT] (10) in K^+^ solution. (**E**) Basket-type form observed for d[(GGGTTA)_3_GGGT] in K^+^ solution (56). *Anti* guanines are colored cyan; *syn* guanines are colored magenta; loops are colored red. M, N and W represent medium, narrow and wide grooves, respectively. Figure reprinted with permission from (10).

The vertebrate telomeric sequence (TTAGGG)_n_ is of special interest, because it is 3–20 kb long in human cells with a ∼200 base overhang of the G-rich 3′-end. This sequence is common to all vertebrates (4). Moreover, even beyond vertebrates, organisms with linear chromosomes have telomeres which contain in general a repetitive sequence capable of forming G-quadruplexes (9). Thus, a biological function of the quadruplex structure in telomeres is highly plausible. The structural properties of many different G-quadruplex DNA-forming sequences have been investigated *in vitro*, applying nuclear magnetic resonance and X-ray crystallography (Figure 1) (10,11). The mammalian telomeric sequence GGG(TTAGGG)_3_ can adopt at least five different structures (10): (i) the basket form, with antiparallel orientation of the strands, observed in solution with Na^+^; (ii) the parallel propeller form, observed in the presence of K^+^ in crystals; (iii+iv) the two (3+1) forms with three parallel strands, observed with K^+^ in solution and (v) a basket conformation with only two base quartets, observed with K^+^ in solution. The predominant form varies with salt conditions (presence of Na^+^ or K^+^), and the nucleotides added at either end (10,12). The different topological forms co-exist in dynamic equilibria; the energy barrier between (3+1) and basket forms is only about 2 kcal mol^−1^ (13). If longer sequences like (TTAGGG)_12_ are studied, the level of complexity increases through combination of the different topologies and stacking interactions of neighboring quadruplexes (14,15).

*In vivo*, the telomeric sequences are ‘capped’, a term used to collectively describe that they are protected from exonucleolytic attack by a combination of protein coverage, and possibly alternative structures that protect the single strand (ss) overhang, such as quadruplex (G4) and/or t-loops. Proteins found at the telomeres include the (mammalian) shelterin complex and the (mammalian and yeast) Cdc13-Stn1-Ten1 (CST) complex. In yeast, CST component Cdc13 (homologue of human POT1) binds to the G-tail and is essential for telomere capping. A temperature-sensitive Cdc13 mutant allows much more exonucleolytic recession of the C-rich strand and thus much longer guanine-rich ssDNA overhangs, which results in activation of the G2/M checkpoint arrest. The phenotype could be recovered by overexpression of different G4-binding proteins, knockout of the G4-DNA-unwinding helicase Sgs1 or addition of small molecule quadruplex ligands (16). All of this would be consistent with G4 helping to rescue this phenotype of extended ss overhangs—directly or indirectly. The authors conclude that G4 DNA can, at least sometimes, be of net benefit. Cdc 13, POT1 and several other proteins binding to G4 sequences (e.g. WRN, BLM, FANCJ and Pif1 helicases and RPA) are reported to unfold the G4 DNA *in vitro* (17–21). G4-stabilizing proteins have also been reported and include Topo I, Nucleolin and MutSα (22,23). Also, the number of mammalian proteins reported to bind to G-quadruplexes *in vitro* is rapidly increasing (23). Recent work also gives more credence to the possible involvement of quadruplexes during transcription and DNA replication (2,24).

Specific and easy to detect quadruplex binding agents would be a valuable and versatile tool to investigate the existence, formation and biological relevance of quadruplex DNA. Many groups have reported the successful synthesis of quadruplex-binding small molecules (25). While these small ligands are very specific for quadruplex DNA as compared to double-stranded DNA, most of them do not, or only weakly, differentiate between different quadruplex folds (26). This lies in the nature of their interactions, which rely mostly on the stacking of suitable planar ring systems to the base quartets. Ligands exhibiting specificity for a particular quadruplex are rare; the acyclic heteroaryle TOxaPy and N-methyl mesoporphyrin IX (NMM) are two promising examples with moderate affinities of 200 nM and 10 μM (27,28). It has also been reported that some displace natural G4-binding proteins (16,29).

In ciliated protozoa such as *Stylonychia lemnae*, extensive DNA reorganization and DNA elimination processes take place in the course of macronuclear differentiation, leading to a macronuclear genome that consists of several millions of gene-sized chromosomes (30,31) with a correspondingly high number of telomers. It has been suggested that quadruplexes can serve to mediate end-to-end association of the macronuclear chromosomes. TEBPα and TEBPβ have been suggested to promote telomere quadruplex formation: knockdown of TEBPα or TEBPβ abolished the signals observed with G-quadruplex specific recombinant antibody scFv fragments (32–34). These antibody scFv fragments have been obtained previously in our laboratory by ribosome display selection from a fully synthetic library by selection on synthetic G-quadruplex DNA with the protozoan sequence (33).

Besides the recombinant antibody scFv fragments generated against the *S. lemnae* telomers (33), G-quadruplex binding scFv fragments and zinc finger proteins have been reported (35,36). scFv fragments selected against human telomeric quadruplex (TTAGGG)_n_ have been used as a probe to detect G-quadruplex formation in fixed cells (37). The authors of this study report punctuate nuclear staining and conclude that the antibodies may detect G-quadruplex-rich regions of unknown origin. However, the antibodies did not discriminate between different G-quadruplex-forming sequences and did not co-localize with the telomeres in immunofluorescence studies. In another study, monoclonal antibodies raised against G4 sequences derived from mammalian and *Oxytricha* telomeres exhibited a granular staining pattern in the nucleus and in metaphase spreads of human cell lines. These signals were abolished through DNAse treatment and thus could be assigned to staining of DNA (38).

We report here the selection of conformation-specific G-quadruplex-binding proteins from libraries of designed ankyrin repeat protein (DARPins) (39). They are highly specific for particular G-quadruplex folds. In contrast to antibodies and zinc finger proteins they contain no cysteines and are therefore not oxidation-sensitive. They can be expressed and they fold in the cytoplasm of any cell, and their high affinity and outstanding biophysical properties make them ideal tools for *in vivo* studies of the formation of specific quadruplexes.

## MATERIALS AND METHODS

### Annealing of oligonucleotides

DNA oligonucleotides were purchased from Microsynth (Switzerland) in PAGE-purified quality. For immobilization on neutravidin-coated surfaces (for ribosome display, enzyme-linked immunosorbent assay (ELISA), or surface plasmon resonance (SPR)), 5′-biotin labeled DNA with a tetra-ethyleneglycol linker was used. All oligonucleotides were dissolved in water and annealed in TBS (50 mM Tris-HCl, 150 mM NaCl, pH 6.8 at room temperature) or TBS-KCl (TBS with 150 mM KCl instead of NaCl) by heating to 95°C and slowly cooling down to 20°C over 1 h in a polymerase chain reaction cycler. Sequences; *tel*, 5′-biotinylated sequence, (TTAGGG)_4_; *tellong*, (TTAGGG)_12_; *teltt*, (TTAGGG)_4_TT; *telcomp*, (CCCTAA)_4_ (mixed in equimolar ratio with *tel* for annealing to obtain dsDNA),

*unspec*, TATGACAACGATCGGAGTACCGAA; *RET*, TAGGGGCGGGGCGGGGCGGGGGCG;

*Hif-1α*, GCGGGGAGGGGAGAGGGGGCGGGAG; *VEGF*, CCGGGGCGGGCCGGGGGCGGGGTC; *c-KIT1*, GAGGGAGGGCGCTGGGAGGAGGGGGCT; *c-KIT2*, CCGGGCGGGCGCGAGGGAGGGGAG; *ILPR*, CAGGGGTGTGGGGACAGGGGTGTGGGGAC; *c-MYC*, TGAGGGTGGGTAGGGTGGGTAA.

### Ribosome display

Ribosome display selections were carried out over three rounds basically according to the standard procedure (40). The 5′-biotinylated target DNA was immobilized on Maxisorp™ 96-well plates (Nunc, Denmark) coated with 66 nM neutravidin (Pierce, USA). Deviating from the standard protocol, the ternary complexes of the stalled ribosomes were buffer-exchanged over NAP-5™ columns (GE Healthcare, USA) into WBT [50 mM Tris–acetate (pH 7.6), 150 mM NaCl, 50 mM Mg(CH_3_COO^−^)_2_ and 0.01% Tween-20] or WBT-KCl [WBT, NaCl substituted with 150 mM KCl] after *in vitro* translation. Thus, selections with essentially only NaCl or KCl could be performed, providing suitable conditions to maintain the salt-dependent folds of the quadruplexes. Panning was performed in WBT or WBT-KCl with increasing stringency for each round. In the third round, 0.8 μM dsDNA (*tel* annealed with *telcomp*) was added as competitor. mRNA was recovered with elution buffer [50 mM Tris–acetate (pH 7.6), 150 mM NaCl and 250 mM ethylenediaminetetraacetic acid] and used for reverse transcription to start the next round.

### Expression and purification of DARPins

All DARPins were expressed in *E. coli* XL1-Blue (Stratagene, acquired by Agilent, USA). The enriched DARPin libraries were cloned into a pQE30-derived *Escherichia coli* expression vector behind an N-terminal MRGSH_6_ tag. DARPins were expressed in 96-well format (41) or in 100 ml terrific broth medium in shake flasks. Four hours after induction with 0.5 mM isopropyl-β-D-thiogalactopyranoside (IPTG) cells were harvested by centrifugation, resuspended in IMAC loading buffer [50 mM Tris, 400 mM NaCl, 20 mM imidazole and 10% (v/v) glycerol, pH 7.4] and sonified. The lysate was cleared by centrifugation at 27,000 × g for 1 h at 4°C. Purification was done on Ni^2+^-nitrilotriacetic acid agarose (NTA-agarose) columns (Qiagen, USA). After washing with IMAC loading buffer, the bound DARPins were eluted by IMAC elution buffer containing 250 mM imidazole. The purity of the DARPins was assessed on 15% sodium dodecyl sulphate-polyacrylamide gel electrophoresis. The eluted samples were loaded on NAP-5™ columns (GE Healthcare, USA) to change buffer conditions to TBS or TBS-KCl. Some DARPins showed limited solubility in TBS or TBS-KCl. In these cases, the eluted samples were centrifuged at 20,000 × g for 15 min at 4°C and the supernatant was used.

### SEC-MALS

The mass and oligomerization state of each DARPin was determined on a liquid chromatography system (AgilentLC1100, Agilent Technologies, Santa Clara, CA, USA) coupled to an Optilab rEX refractometer (Wyatt Technology, Santa Barbara, CA, USA) and a miniDAWN three-angle light-scattering detector (Wyatt Technology). A 24 ml Superdex 200 10/30 column (GE Healthcare Biosciences, Pittsburg, PA, USA) was run at 0.5 ml/min in TBS and TBS-KCl for protein separation. Data were analyzed with the ASTRA software (version 6.0.1.10; Wyatt Technology).

### ELISA experiments

All steps for ELISA tests were performed at ambient temperature in TBS and TBS-KCl with 0.05% (v/v) Tween-20. 5′-biotinylated DNA (100 nM) was coated via neutravidin for 1 h. IMAC-purified DARPins (50 nM) or 1:10 diluted crude extracts were incubated for 40 min. An anti RGS-His antibody (Qiagen, Germany) and an anti-mouse antibody alkaline phosphatase conjugate (Sigma) were used for detection.

### SPR studies

SPR measurements were performed on a ProteOn XPR36 instrument (Biorad). Biotinylated, annealed oligonucleotide (300–500 RU) was immobilized on a NeutrAvidin NLC sensor chip (Biorad). The sensor chip was then undocked, rinsed with ddH_2_O, air-dried and re-inserted. This procedure was performed to bring the surface in a condition to obtain stable results. IMAC-purified DARPins (1 nM to 32 nM) were injected at a flow rate of 100 μl/min for 240 s. Dissociation was followed over at least 600 s. Simple Langmuir kinetic fitting was applied where appropriate with the ProteOn Manager software.

To determine the *K_D_* in solution, competition SPR was carried out. For this purpose 32 nM DARPin was incubated with 0, 16, 32, 64, …, 1024 nM *tel* DNA (non-biotinylated) as competitor. The samples were injected onto the *tel*-coated surface under the conditions described above. Since under the experimental conditions, the system reached equilibrium binding, but kinetics were not monophasic, the binding plateaus were used to evaluate inhibition. Thus, the measured response units (RU) at equilibrium were taken as a measure for the remaining concentration of free DARPin in the samples. However, the measured RU are not linear with free DARPin, and thus injections of 4, 8, 16 and 32 nM of each DARPin without competitor were performed to produce standard curves of RU versus DARPin concentration in solution, *B*_sol_. Fitting was done with two parameters (*a* and *b*) according to Equation (1):

(1) \begin{equation*} {\rm RU} = \frac{{a \times B_{\rm sol} }}{{b + B_{{\rm sol}} }} \end{equation*}

Solving for *B*_sol_ yields

(2) \begin{equation*} B_{\rm sol} = \frac{{b \times \rm RU}}{{a - \rm RU}} \end{equation*}

where *B*_sol_ represents the DARPin concentration in solution as a function of measured RU.

*K_D_* was then fitted from the competition data. The equation for the fit was developed as follows: *K_D_* is defined as:

(3) \begin{equation*} K_D = \frac{{A_{{\rm free}} \times B_{{\rm free}} }}{{AB}} \end{equation*}

*A*_free_ and *B*_free_ are the (unknown) free concentrations of DNA and DARPin, respectively, and *AB* is the concentration of the complex at equilibrium. In combination with the law of conservation of mass, Equation (4) is obtained (where *A*_tot_ and *B*_tot_ are the total concentrations of DNA and DARPin):

(4) \begin{equation*} K_D = \frac{{(A_{{\rm tot}} - AB) \times (B_{{\rm tot}} - AB)}}{{AB}} \end{equation*}

If Equation (4) is solved for *AB* and combined with Equation (5),

(5) \begin{equation*} B_{{\rm free}} = B_{{\rm tot}} - AB \end{equation*}

Equation (6) is obtained:

(6) \begin{eqnarray*} &&B_{{\rm free}} = B_{{\rm tot}} - \nonumber \\ &&\frac{{K_D{+}A_{{\rm tot}}{+}B_{{\rm tot}}{-}\sqrt {({-}K_D{-}A_{{\rm tot}}{-}B_{{\rm tot}} )^2 {-}4{\times}A_{{\rm tot}}{\times}B_{{\rm tot}} } }}{2} \nonumber \\ \end{eqnarray*}

Equation (6) was used to fit the competition data with SigmaPlot, where *B*_free_ was taken from the measured RU using Equation (2) (using *B*_sol_ = *B*_free_). The fit was performed globally over all injections of DARPin with different concentrations of competitor DNA.

### CD spectroscopy

CD measurements were performed with a Jasco J 810 Polarimeter at 22°C in cuvettes with 1 mm path length. Quadruplex-forming DNA oligos were folded in TBS or TBS-KCl as described above. Protein:DNA complexes were allowed to form for at least 30 min before the measurement was started. The instrument settings were: 50 nm/min scan speed, 4 s integration time, 2 nm band width. Each spectrum was accumulated three times and averaged.

## RESULTS

### Selection of G-quadruplex binding DARPins and primary screening

Two different quadruplex targets were used to select binders: the sequence (TTAGGG)_4_TT can form one quadruplex unit with different topologies (Figure 1), (10), while (TTAGGG)_12_ may form up to three quadruplex units, including compact forms with two or three contiguous quadruplexes (14,15). Folding of quadruplex structures was tested with CD spectroscopy for both sequences. The obtained CD-spectra (data not shown, spectra were similar to those of (TTAGGG)_4_, shown as black line with small circles in Figure 4B and C) were in accordance with antiparallel conformation in NaCl, and (3+1) forms in KCl. Also antiparallel and propeller conformations may be populated to some extent in the K^+^-containing buffer (12). Selections were carried out with KCl- and NaCl-containing buffers in parallel to include a larger portion of the conformational target space and to produce conformation-specific binders.

**Figure 2.**
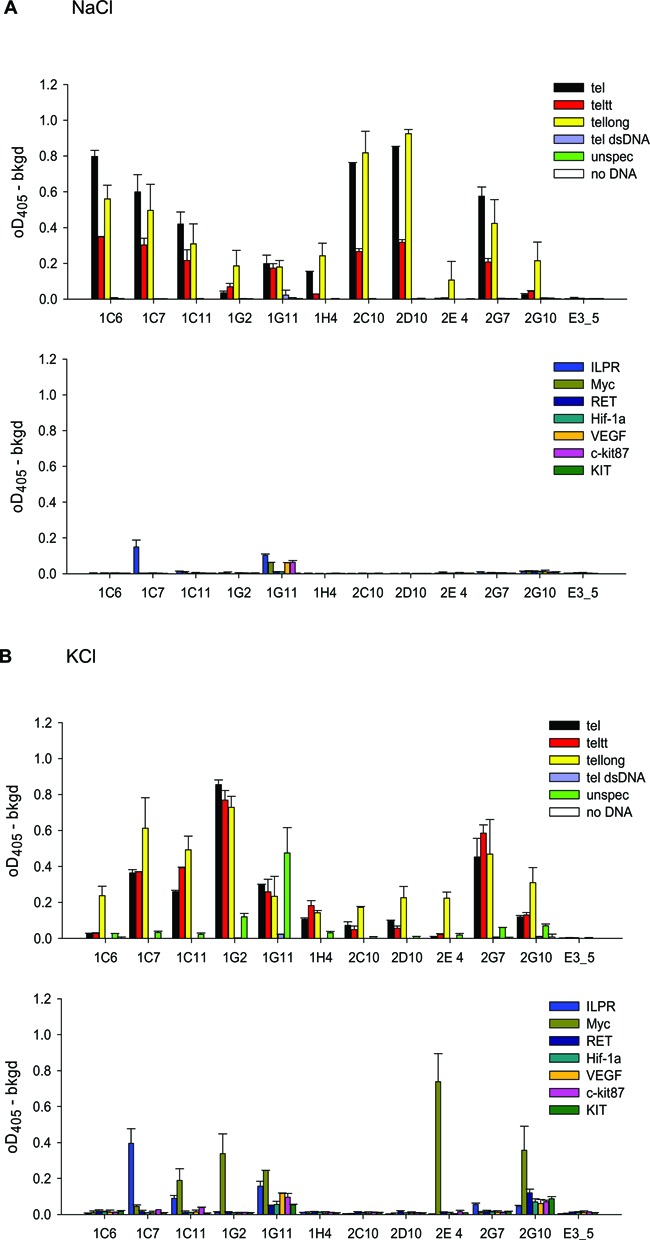
ELISA with 100 nM immobilized DNA targets and 50 nM DARPins. The experiment was performed in TBS with 150 mM NaCl (**A**) and TBS with 150 mM KCl (**B**). Most DARPins specifically bind the telomere sequences. Variants 1G11 and 2G10 have a relaxed specificity for different quadruplexes. DARPin E3_5 was not selected for DNA binding and served as a negative control.

**Figure 3.**
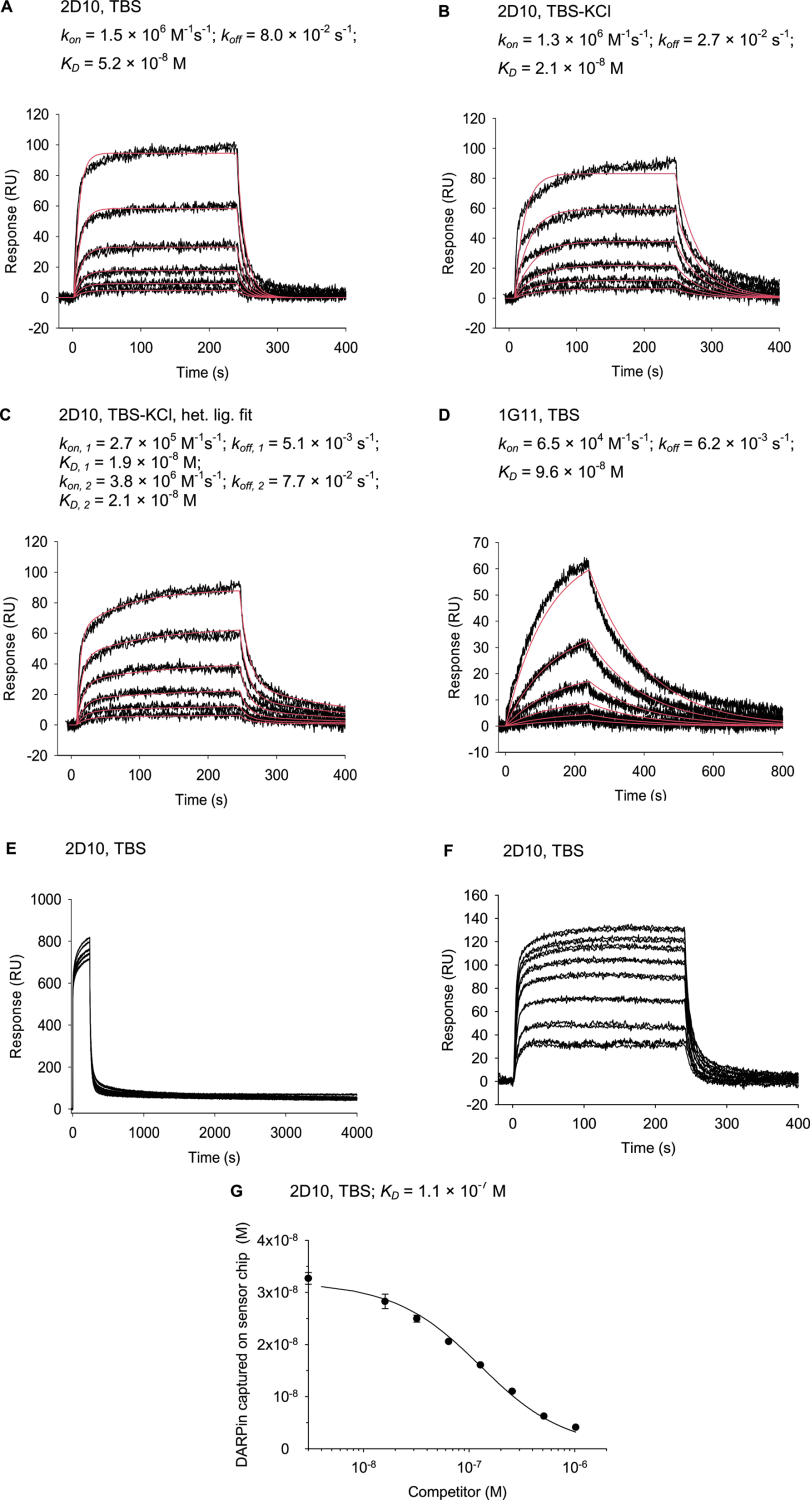
Typical SPR data obtained with *tel* DNA, representing the different binding behaviors found. (**A**) Kinetic fit of 1, 2, 4, 8, 16, 32 nM injections of 2D10 recorded in TBS and (**B**) in TBS-KCl. (**C**) Dataset from (B), fitted with heterogeneous ligand model. (**D**) Kinetic fit of 1, 2, 4, 8, 16, 32 nM injections of 1G11 (which has a dimeric fraction) recorded in TBS. (**E**) Injection of DARPins at higher concentrations (1.5, 3, 6,12 μM) leads to saturation of the sensorchip surface, shown for 2D10. (**F**) Examples of sensorgrams obtained in a competition setup with 32 nM 2D10 and 0, 16, 32, 64,…, 1024 nM *tel* competitor. (**G**) Plateau values from (F) as a function of inhibitor concentration to measure for free DARPin concentrations at equilibrium. The fit using Equation (6) is shown.

**Figure 4.**
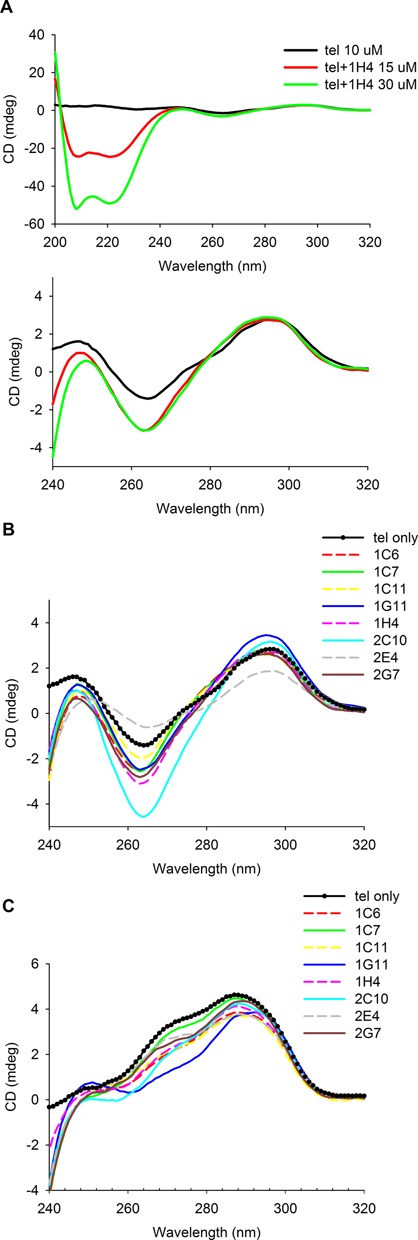
CD spectra of *tel* alone and in complex with DARPins. (**A**) Upper panel (full spectrum, dominated by protein): 10 μM *tel* in TBS before (black line) and after addition of 15 μM (red line) and 30 μM (green line) DARPin 1H4. Lower panel (zoom into nucleotide region): same data, detailed view from 240 nm to 320 nm. Between 250 nm and 320 nm, increase of protein concentration from 15 μM to 30 μM did not change the signal arising from the DNA quadruplex, indicating saturation of the complex. (**B**) 10 μM *tel* in TBS (Na^+^) (dotted line) and complexed with 15 μM DARPins. (**C**) Same experiment carried out in TBS-KCl.

The selection was performed in three rounds of ribosome display from two libraries (N2C and N3C) of DARPins (39). These abbreviations denote either two or three internal ankyrin repeats with randomized residues between an N- and C-capping repeat. So far, no DNA-binding ankyrin has been reported, and no binding to DNA was observed with unselected DARPins, which by design have a rather acidic pI. We found it important to use proteins without any known DNA-binding activity to minimize the chance to obtain binders with intrinsic affinity to dsDNA. To further remove potential dsDNA binders, the telomere sequence, hybridized to its complementary strand, was added in double-stranded format as a competitor to the third selection round.

After round three, the enriched DNA pools were cloned and expressed in *E. coli*. ELISA screening of crude extracts from 200 clones with immobilized DNA revealed 20 binders. All 20 binders were purified by a single IMAC step and screened by SEC-MALS for their oligomerization state. Only DARPins 1H4 and 1G11 showed a dimeric portion, all others were monomeric. No hints for soluble aggregates could be detected. The 11 best binders (10 from the N3C library, 1 from the N2C library) (Supplementary Table ST1) were chosen for further characterization. All sequences were unique. The randomized positions show a preference for positively charged residues: when considering only the randomized residues, of 32 randomized repeats in the selected DARPins, 14 have a positive net charge, 13 are neutral and only 5 show a negative net charge. Considering the charge over the whole protein, seven binders have an overall positive charge, compared to one neutral and three negatively charged binders.

### Specificity of selected DARPins in ELISA

ELISA results for the best 11 binders are shown in Figure 2. To investigate the obtained candidates for their ability to discriminate between different quadruplex folds, additional quadruplex-forming DNA sequences were used. We have chosen seven well-described sequences from human promoter regions: the *RET*, *HIF-1α*, *VEGF*, *c-KIT1*, *c-KIT2*, *ILPR* and *c-MYC* sequences (11,42,47,57). The assay was performed in normal Na^+^-containing TBS and in TBS-KCl (where NaCl has been substituted by KCl) to probe the cation-dependent conformations of the telomere sequences or influence of different primary sequence on quadruplex formation. This cation dependence is of interest, since the mammalian cell contains of course much higher concentrations of K^+^ than Na^+^.

Discrimination between the NaCl and KCl forms of the telomere targets was observed: DARPin 1G2 gave higher ELISA signals in TBS-KCl, while 2C10 and 2D10 gave higher signals in Na^+^-containing TBS. The DARPins gave also distinct signals with the three telomeric sequences (TTAGGG)_12_, (TTAGGG)_4_ and (TTAGGG)_4_TT. Some DARPins recognized only the (TTAGGG)_12_ sequence (e.g. 2E4, 2G10 in TBS and 2E4, 1C6 in TBS-KCl). This implies that a unique structural feature is present exclusively in the longer sequence and this is recognized by these particular DARPins. This sequence has previously been reported to be able to form a compact array of quadruplexes (14), in addition to separated quadruplex units arranged like beads on a string. In TBS, (TTAGGG)_4_ often led to higher signals than found for (TTAGGG)_4_TT for some DARPins (1C7, 1C6, 2G7, 2D10, 2C10), indicating that structural differences between (3+1) form 1 and form 2 (Figure 1C and D) are picked up by the different DARPins (10). Both sequences, (TTAGGG)_4_ and (TTAGGG)_4_TT, yielded the same signal with 1G11, a binder with relaxed specificity (see below).

Many of the selected DARPins were highly specific for the telomeric sequences and did not bind to the other quadruplexes. However, two of the DARPins, 1G11 and 2G10, showed a relaxed sequence specificity and recognized several quadruplexes. DARPins 1C7, 1C11, 1G2 and 2E4 recognized the *ILPR* or *c-MYC* sequence in addition to the telomere sequence, partly depending on the buffer used. Consequently, the DARPins are able to discriminate structural features which are different in each quadruplex, like loop length, loop sequence and structure or different groove sizes. In contrast, variants 1G11 and 2G10 seem to recognize a common structural element (i.e. a common epitope) of G-quadruplexes. Interestingly, 1G11 additionally gives signals with the *unspec* DNA oligonucleotide, but not with *ds-tel*, thus excluding a completely non-specific interaction with any DNA. In summary, a repertoire of specificities for different quadruplex forms and sequences was obtained among the selected DARPins.

### SPR studies

Affinities of the quadruplex-binding DARPins were quantified by SPR in Na^+^- and K^+^-containing buffers, applying the different biotinylated oligonucleotide as ligand on a neutravidin-coated sensor chip and injecting the proteins as analytes. Two variants of the telomere sequence, *tel* and *teltt*, were used for the SPR studies. The two additional 3′-thymidines in *teltt* shift the predominant form from (3+1) form 1 to form 2 (Figure 1C and D) (43). Because the results obtained with *teltt* are essentially equal to those obtained with *tel*, only the latter are shown. The *ds-tel* DNA and the *unspec* oligonucleotides with an unrelated sequence were used as controls and produced no or little response in all cases. Data sets recorded in Na^+^-containing TBS could be fitted with a simple Langmuir model reasonably well, if protein concentrations from 1 nM to 32 nM were used (see Figure 3 for typical data, Table 1 for *K_D_* values; Supplementary Table ST2 and Figures S1–S3 for rate constants).

**Table 1.** *K_D_* values obtained with SPR in TBS

	*tel*	*tel*	*ILPR*	*c-MYC*
DARPin variant	*K_D_* from kinetics (nM)	*K_D_* from competition (nM)	*K_D_* from kinetics (nM)	*K_D_* from kinetics (nM)
1C6	16 ± 3	56 ± 3	nb	nb
1C7	37 ± 15	110 ± 10	33 ± 8	81 ± 38
1C11	53 ± 22	100 ± 5	43 ± 29	49 ± 27
1G2	53 ± 44	69 ± 7	27 ± 9	43 ± 19
1G11	72 ± 46	^a^	44 ± 30	19 ± 3
1H4	62 ± 34	81 ± 7	nb	nb
2C10	48 ± 12	67 ± 5	nb	nb
2D10	34 ± 16	110 ± 10	nb	nb
2E4	nb	nb	nb	64 ± 40
2G7	20 ± 3	32 ± 2	nb	nb
2G10	nb	nb	nb	90 ± 59

nb, no binding, i.e. no or very weak RU signal.

^a^Complex behavior, could not be determined, see text.

On the other hand, for datasets recorded in TBS-KCl, a model with ligand heterogeneity was more appropriate, which is discussed below as reflecting the pre-existing structural heterogeneity of the G-quadruplexes, of which one conformer seems to be preferentially bound. Depending on the epitope of the DARPin, it detects more or less of this heterogeneity in the target, while the affinity itself may be responsible to which extent binding can drive the heterogeneous ligand conformation into a homogeneous complex. The *K_D_* values determined for the first and second binding event differed between 2-fold and 10-fold (Table 2, Supplementary Table ST3 and Figures S4–S6).

**Table 2.** *K_D_* values obtained with SPR in TBS-KCl

	*tel*		*tel*	*ILPR*		*c-MYC*	
DARPin variant	*K_D_* from kinetics (nM)		*K_D_* from competition (nM)	*K_D_* from kinetics (nM)		*K_D_* from kinetics (nM)	
	First equil.	Second equil.		First equil.	Second equil.	First equil.	Second equil.
1C6	nb	nb	nb	nb	nb	nb	nb
1C7	15 ± 1	3.3 ± 3.0	160 ± 11	21 ± 2	14 ± 8	^a^	^a^
1C11	11 ± 2	8.9 ± 8.3	72 ± 4	12 ± 2	4.0 ± 4.6	17 ± 3	6.8 ± 4.3
1G2	7.9 ± 1.9	3.3 ± 1.1	3.6 ± 2.0	9.4 ± 3.2	6.4 ±7.4	16 ± 5	14 ± 4
1G11^a^	^a^	^a^	^a^	^a^	^a^	^a^	^a^
1H4	15 ± 4	14 ± 7	19 ± 2	nb	nb	nb	nb
2C10	20 ± 1	35 ± 28	160 ± 17	nb	nb	nb	nb
2D10	18 ± 3	22 ± 2	160 ± 10	nb	nb	nb	nb
2E4	4.6 ± 4.7	42 ± 48	120 ± 10	nb	nb	28 ± 13	19 ± 27
2G7	10 ± 5	5.5 ± 1.9	8.0 ± 2.0	nb	nb	nb	nb
2G10	65 ± 69	15 ± 11	nb	22 ± 5	19 ± 24	100 ± 150	15 ± 5

nb: no binding, i.e. no or very weak RU signal.

^a^Complex behavior, could not be determined, see text.

Typical *K_D_* values observed at 1–32 nM DARPin concentration range from 10 nM to 100 nM. Higher DARPin concentrations resulted in complex sensorgrams (Figure 3E). If DARPin concentrations above 1 μM were injected, the height of the RU signal only slightly increased further, indicating saturation of the chip surface and absence of non-specific binding. Sensorgrams recorded with such high analyte concentrations contain components with very slow off-rates, the slowest being typically 7 × 10^−5^ s^−1^.

To probe the specificity against different quadruplexes, which had been observed in the ELISA, the *c-MYC* and *ILPR* sequences were also applied as immobilized ligands. The high specificity of DARPins 1H4,2C10, 2D10 and 2G7 could be confirmed, as no or very low RU response was observed with the *c-MYC* and insulin sequences in TBS and TBS-KCl. All samples for which a sufficient signal for *K_D_* calculation was detected are summarized in Tables 1 and 2. The obtained specificity profiles basically confirmed the ELISA results. Especially the recognition of *c-MYC* by 2E4 and *ILPR* by DARPin 1C7 could be confirmed. DARPin–DNA combinations with no ELISA signal gave mostly no SPR signal as well. However, both assays explore different characteristics of the binders: the standard ELISA protocol includes ∼2 h time for the DARPin–DNA complex to equilibrate (i.e. incubation with detection antibodies and washing steps) and thus detects predominantly slow off-rate binding events, after the DNA in the complex had a long time to reach an equilibrium conformation. The SPR protocol, in contrast, was designed to quantify affinity at low nanomolar concentrations of DARPin using a faster timescale of 240 s injection and 600 s dissociation time. Thus, concordant results are not necessarily expected, since in this timeframe conformers may not necessarily reach equilibrium, and both methods rather complement each other in the information they can give about the system.

SPR competition experiments were carried out with the *tel* sequence to further confirm the obtained *K_D_* values and to probe the specificity of the interaction in solution (see Figure 3F and G for typical data). All SPR signals could be competed through an excess of free *tel* quadruplex and fitted with a one-site model (see the Materials and Methods section). In general, good accordance of the *K_D_* values obtained from direct and competition SPR measurements was observed in Na^+^-containing TBS. *K_D_* values calculated from the competition experiments in TBS-KCl, on the other hand, were often up to one order of magnitude higher than the corresponding values from direct measurements. This difference may reflect the heterogeneity of DNA conformations in the presence of potassium and will be discussed below. DARPins 1H4 and 2G7 revealed the same *K_D_* in TBS-KCl for both measurement methods, and thus may recognize an epitope common to both conformations or very efficiently drive the equilibrium to one conformation, further confirming the intrinsic comparability of the methods. In the competition setup, the best *K_D_* of 3.6 ± 2 × 10^−9^ M was measured for 1G2 and *tel*.

Interestingly, the sensorgrams obtained with 1G11 in TBS are distinct from all others through slower association and dissociation kinetics (Figure 3D). 1G11 has a dimeric fraction, and it is possible that the observed kinetics are a sum of monovalent and bivalent binding. Bivalent binding would require that the dimeric fraction of 1G11 can make bivalent contacts to the immobilized DNA. This is reminiscent of similar observations with multimeric miniantibodies, where this phenomenon has been studied (44,45). In the competition test, the lowest concentration of competitor (16 nM) was already sufficient to almost completely prevent 1G11 (32 nM) from binding to the sensor chip, as would be expected for a fast equilibrating system with two binding sites, where the tight interaction is a consequence of bivalent binding (44,45). In TBS-KCl, all sensorgrams with 1G11 and the combination 1C7/*c-MYC* showed complex shape, precluding reasonable fits. This can be interpreted as an overlay of many binding events with different affinity and different kinetics.

### CD spectroscopy studies of DARPin–DNA complexes

CD measurements were carried out with the *tel* sequence at 10 μM DNA concentration, which is 100-fold to 3000-fold above *K_D_*. Saturation of DNA with protein was confirmed by application of DARPin 1H4 in two different concentrations, namely 15 μM and 30 μM. While the CD signal of the protein (200–240 nm) increases accordingly, the DNA CD signal between 250 and 300 nm is the same for both concentrations, indicating complete complex formation. No hints for unfolding of the quadruplex are seen.

On the contrary, in the presence of sodium chloride, most DARPins and particularly 2C10 led to an increase of amplitude for the negative 260 nm signal and the positive 295 mn signal, suggesting a stabilization of the existing basket conformation (Figures 1A and 4B). Only 2E4 seemed to weaken the structure, as the decreased signal amplitudes would suggest. It should also be noted that 2E4 binds only *tellong* and *c-MYC* in the ELISA. Thus, it may recognize the parallel propeller conformation of *c-MYC* in K^+^ containing buffers (Figure 1B) and structures only present in *tellong*. The 300-fold higher concentrations used for the CD measurement seemed to force binding and deformation of the quadruplex.

The CD signal of *tel* in K^+^-containing buffers is caused by the (3+1) conformations (Figure 1C and D). Addition of the DARPins led to a decrease in ellipticity primarily around 270 nm, most pronounced for 1G11 (Figure 4C). The most likely interpretation is that the DARPins recognize their epitopes on the (3+1) conformation, but alter the conformation due to an induced fit mechanism or through conformational selection. These data do not allow us to distinguish whether the (3+1) conformation is altered or whether a mixed population is induced, containing the (3+1) conformation together with molecules in a basket-like conformation (as they are present in the Na^+^ complexes).

Our conclusion from the CD measurements is that all tested DARPins except for 2E4 bind and stabilize the basket conformation in Na^+^-containing buffer, while they alter or distort the (3+1) conformation in K^+^-containing buffer.

## DISCUSSION

We could select DARPin binders that specifically recognize the quadruplexes formed by human telomeric DNA. More importantly, the different DARPins can distinguish the different forms of the quadruplex, depending on conformation and/or primary sequence. These different conformations are favored, depending on the one hand by the different monovalent metal ions present, on the other hand by the total length or the single-stranded DNA, in turn determining the degree of stacking. The presence of Na^+^ or K^+^ influences which direction of assembly of the four strands is energetically favored and thus also determines the conformation of the loops connecting them. Some of the DARPins bind exclusively to the telomere sequence, while others bind, in addition, to other quadruplex-forming sequences tested, like *ILPR* or *c-MYC*.

The CD data of the DARPin–*tel* complexes clearly show that DARPins 1C6, 1C7, 1C11, 1G11, 1H4, 2C10 and 2G7 prefer and stabilize the antiparallel basket form, since the spectral features are not changed. In Na^+^ solutions, this form is predominant anyway in the absence of DARPins. In K^+^ solutions the basket together with the parallel propeller form is only present at low levels, as most of the population is in the dominating (3+1) forms. The DARPins appear to deform the (3+1) forms and/or to shift the equilibrium somewhat toward the basket form, as evidenced by the appearance of CD features consistent with the basket form.

The complexity of many sensorgrams obtained in the SPR experiments reflects the properties of the target molecules: quadruplex DNA presents many similar, but not identical surface features (10) (cf. Figure 1): grooves consisting of the same sequence, but of different widths (caused by *syn* or *anti* glycosidic conformations) and different accessibility (some grooves are covered by loops) and loops with the same sequence (in telomeric sequences), but different conformation (edgewise, diagonal or double-chain-reversal). Furthermore, the planar surface of the terminal base quartets may be covered by loops to a degree which varies with *syn* or *anti* glycosidic conformation. Consequently, any epitope consisting of one or more of these surface features will be present in slightly different versions. The conformational heterogeneity of the vertebrate telomere sequence in K^+^-containing buffers increases once again the number of surface features that may be simultaneously present. The consequence of this complexity in K^+^ buffers is that an overlay of binding events with different *K_D_* is measured.

While SPR curves recorded in Na^+^-containing TBS could be approximated with simple Langmuir kinetics (for an example, see Figure 3A), the SPR curves in K^+^ could not, and they were thus fitted with a heterogeneous ligand model (see, e.g. Figure 3B and C). Interestingly, two rather similar *K_D_* values resulted, one with fast and the other with slow kinetics. One possible explanation is that a fraction of the molecules is already present in the conformation that is recognized (e.g. (3+1) form 1 or basket form), while the other fraction (e.g. (3+1) form 2) has to undergo a conformational change to arrive at the state being recognized. We currently have no further insight whether this would happen through a model of conformational selection or induced fit under these conditions (46).

In TBS, a Na^+^-containing buffer, the values obtained in solution (from competition SPR) and on the sensor chip surface agree very well (Tables 1 and 2), in other cases in TBS-KCl, a difference of up to a factor 10 is observed between direct and competition measurements. This is undoubtedly caused by the different DNA conformations. Since the *K_D_* in the inhibition experiment is always related to the *total* DNA concentration, a smaller percentage of the relevant conformer translates into an actual concentration of the inhibitor employed lower than presumed, and thus an apparent affinity worse than the true one. Consequently, affinities deduced from competition SPR were most likely too low. Additionally, conformations may have different preferences in solution and when immobilized. Importantly, the values in TBS agree between the two methods, emphasizing that the methodology per se is robust, consistent with a preferential conformation being present in Na^+^ buffers.

ELISA and SPR studies showed in general similar binding profiles for the DARPins for a range of quadruplex-forming sequences, which also emphasizes that the methods are solid. (3+1) forms in KCl may be recognized better by some of the DARPins on a fast timescale, as the trend to lower *K_D_* values (higher affinities) in the SPR results implies, which predominantly measures short-time behavior. In the ELISA, only 1G2 gave higher signals with TBS-KCl than in TBS (Na^+^) and the telomere oligos. This may again reflect the conformational heterogeneity: switching between conformations, which is possible because of the low energy barrier of only 2 kcal mol^−1^ (13), may lead to loss of the DARPins, which are then washed away in the ELISA.

The fact that different DARPins have different sequence preferences shows that indeed the different DARPins do recognize distinct epitopes, which are present to different degrees in the various oligonucleotides and under different conditions, notably the type of alkali cation present. Most of the DARPins, which are specific for the telomere quadruplex and do not recognize any of the other sequences, must definitely bind to a structure unique to this target. Conversely, variants like 1G11 and 2G10 appear to recognize structural features common to all investigated G4 forms.

Some of the DARPins selected against the telomeres cross-react with only one of the other potential quadruplex-forming oligonucleotides. Thus, DARPin 1C7 probably recognizes one epitope shared between the telomere quadruplex and the *ILPR* quadruplex. Structural studies of *ILPR* suggest an antiparallel conformation, which differs from the antiparallel *tel* quadruplex in the loop sequence and by having a fourth G-tetrad in the stack (47). Structural features common to both G4 would then be loop length (and possibly conformation) and the antiparallel orientation with the corresponding groove widths. DARPins 1G2 and 2E4 recognize an epitope shared between the telomere quadruplex and the *c-MYC* structure. The *c-MYC* quadruplex adopts propeller conformation (48) like *RET* and *c-KIT2*, which are not bound, however, by DARPins 1G2 and 2E4. Thus, the common epitope may include, for example, the double-chain-reversal loop structure, which is common to the propeller and (3+1) conformations. In contrast to *RET* and *c-KIT2*, only *c-MYC* contains loops with sequences very similar to the telomere quadruplex. The other G4 sequences which have been tested in the ELISA are not recognized and thus seem to form less related structures. These binding profiles narrow down the potential epitopes and must now be backed up by structural studies to map the actual epitopes recognized by the DARPins.

The preferences for different conformations and for different quadruplex primary sequences among the different DARPins indirectly show that indeed different molecular surfaces of the target are bound and thus differentiated. This feature also provides an invaluable tool for discriminating conformations on a very small scale in binding assays which might eventually approach the single molecule level, since the DARPins can be conveniently fluorescently labeled. Such a sensitive binding assay for conformation can complement other biophysical methods, which require much more material and are thus not suitable for DNA isolated from a cell. This property to distinguish quadruplex conformations and sequences sets the presented DARPins apart from most small molecule binders, which in general exhibit only weak discrimination power between the different types of DNA quadruplexes.

Two questions remain unanswered in the current study: (i) it has to be tested if the DARPins are able to distinguish between RNA and DNA quadruplexes. There is evidence that telomeric DNA is transcribed (49,50) and *in vivo* studies have to consider this finding. (ii) We have tried to visualize the telomeric G-quadruplex in human cells. The telomeres were fluorescently labeled through shelterin-m-Cherry fusions. As the next step we introduced protein fusions of the G4-binding DARPins with GFP. The length of the G-tail allows for formation of ∼8 quadruplex structures per telomere. Thus, very weak signals are to be expected. Consequently, a sufficiently low amount of the ‘DARPin probe’ and/or extensive washing steps are required to avoid flooding the cells with background signal. We could detect spot-like signals in the nuclei with confocal microscopy. However, there was never any satisfactory co-localization with the telomers, and the level of background signal observed with a non-specific DARPin probe was not convincingly different. More extensive studies, preferably with single molecule sensitivity, are required to address the technical challenges and finally collect conclusive and unequivocal *in vivo* data. For other purposes, DARPins have already been successfully applied to study intracellular localization of their targets (51).

More general, G4-binding DARPins can be used as tools to investigate and discriminate structural properties and occurrence of quadruplexes. DARPins can be expressed within bacterial, yeast and mammalian cells, labeled and detected in live cells, to elucidate the biology of quadruplexes. They may thus become an important tool complementing current approaches. Presently, much research on the significance of quadruplex formation in telomere biology and for potential quadruplex-forming sequences within chromosomes in the regulation of gene expression is relying on point mutations in the sequences in question. Especially for these chromosome-internal sequences, frequently the difference in expression between mutants is used as read-out (52). Unfortunately, this has the strong disadvantage that it cannot distinguish between effects caused by DNA conformation and by the primary DNA sequence alone, e.g. through differential recognition of transcription factors or other DNA- or RNA-binding molecules, or through differential RNA degradation, or micro-RNAs encoded in this region, all influencing cell biology without quadruplex formation.

Specific quadruplex-binding proteins like DARPins that can be directly expressed in the cell would allow a more direct approach: they could easily be linked with transcriptional activators in a one-hybrid setup to monitor quadruplex formation *in vivo*. No alterations to the DNA sequence and no external administration of G4 ligands would be necessary. While the detection of quadruplexes in ciliated protozoa with their extremely high number of telomers has been comparatively straightforward (32), the direct detection of fluorescently labeled DARPins binding to quadruplexes in live cells is more challenging, because of the much smaller number of telomers and potential gene regulatory sequences, if specific locations are probed. Nonetheless, progress in advanced high-resolution light microscopy techniques may make such approaches feasible.

## SUPPLEMENTARY DATA

Supplementary Data are available at NAR Online.

SUPPLEMENTARY DATA

## References

[1] Bryan T.M., Baumann P. (2010). G-quadruplexes: from guanine gels to chemotherapeutics. Methods Mol. Biol..

[2] Kaushik M., Kaushik S., Bansal A., Saxena S., Kukreti S. (2011). Structural diversity and specific recognition of four stranded G-quadruplex DNA. Curr. Mol. Med..

[3] Lane A.N., Chaires J.B., Gray R.D., Trent J.O. (2008). Stability and kinetics of G-quadruplex structures. Nucleic Acids Res..

[4] Lipps H.J., Rhodes D. (2009). G-quadruplex structures: in vivo evidence and function. Trends Cell Biol..

[5] Huppert J.L. (2010). Structure, location and interactions of G-quadruplexes. FEBS J..

[6] Bugaut A., Balasubramanian S. (2012). 5′-UTR RNA G-quadruplexes: translation regulation and targeting. Nucleic Acids Res..

[7] Hammond-Kosack M.C., Docherty K. (1992). A consensus repeat sequence from the human insulin gene linked polymorphic region adopts multiple quadruplex DNA structures in vitro. FEBS Lett..

[8] Hammond-Kosack M.C., Dobrinski B., Lurz R., Docherty K., Kilpatrick M.W. (1992). The human insulin gene linked polymorphic region exhibits an altered DNA structure. Nucleic Acids Res..

[9] Huppert J.L. (2008). Four-stranded nucleic acids: structure, function and targeting of G-quadruplexes. Chem. Soc. Rev..

[10] Phan A.T. (2010). Human telomeric G-quadruplex: structures of DNA and RNA sequences. FEBS J..

[11] Qin Y., Hurley L.H. (2008). Structures, folding patterns, and functions of intramolecular DNA G-quadruplexes found in eukaryotic promoter regions. Biochimie.

[12] Singh V., Azarkh M., Exner T.E., Hartig J.S., Drescher M. (2009). Human telomeric quadruplex conformations studied by pulsed EPR. Angew. Chem..

[13] Chaires J.B. (2010). Human telomeric G-quadruplex: thermodynamic and kinetic studies of telomeric quadruplex stability. FEBS J..

[14] Petraccone L., Spink C., Trent J.O., Garbett N.C., Mekmaysy C.S., Giancola C., Chaires J.B. (2011). Structure and stability of higher-order human telomeric quadruplexes. J. Am. Chem. Soc..

[15] Singh V., Azarkh M., Drescher M., Hartig J.S. (2012). Conformations of individual quadruplex units studied in the context of extended human telomeric DNA. Chem. Commun..

[16] Smith J.S., Chen Q., Yatsunyk L.A., Nicoludis J.M., Garcia M.S., Kranaster R., Balasubramanian S., Monchaud D., Teulade-Fichou M.P., Abramowitz L. (2011). Rudimentary G-quadruplex-based telomere capping in Saccharomyces cerevisiae. Nat. Struct. Mol. Biol..

[17] Zaug A.J., Podell E.R., Cech T.R. (2005). Human POT1 disrupts telomeric G-quadruplexes allowing telomerase extension in vitro. Proc. Natl Acad. Sci. U.S.A..

[18] Kamath-Loeb A., Loeb L.A., Fry M. (2012). The Werner syndrome protein is distinguished from the Bloom syndrome protein by its capacity to tightly bind diverse DNA structures. PLoS One.

[19] Wu Y., Sommers J.A., Khan I., de Winter J.P., Brosh R.M. (2012). Biochemical characterization of Warsaw breakage syndrome helicase. J. Biol. Chem..

[20] Sanders C.M. (2010). Human Pif1 helicase is a G-quadruplex DNA-binding protein with G-quadruplex DNA-unwinding activity. Biochem. J..

[21] Salas T.R., Petruseva I., Lavrik O., Bourdoncle A., Mergny J.L., Favre A., Saintome C. (2006). Human replication protein A unfolds telomeric G-quadruplexes. Nucleic Acids Res..

[22] Gonzalez V., Hurley L.H. (2010). The C-terminus of nucleolin promotes the formation of the c-MYC G-quadruplex and inhibits c-MYC promoter activity. Biochemistry.

[23] Wu Y., Brosh R.M. (2010). G-quadruplex nucleic acids and human disease. FEBS J..

[24] Sissi C., Gatto B., Palumbo M. (2011). The evolving world of protein-G-quadruplex recognition: a medicinal chemist's perspective. Biochimie.

[25] Luedtke N.W. (2009). Targeting G-quadruplex DNA with small molecules. Chimia.

[26] Tran P.L., Largy E., Hamon F., Teulade-Fichou M.P., Mergny J.L. (2011). Fluorescence intercalator displacement assay for screening G4 ligands towards a variety of G-quadruplex structures. Biochimie.

[27] Hamon F., Largy E., Guedin-Beaurepaire A., Rouchon-Dagois M., Sidibe A., Monchaud D., Mergny J.L., Riou J.F., Nguyen C.H., Teulade-Fichou M.P. (2011). An acyclic oligoheteroaryle that discriminates strongly between diverse G-quadruplex topologies. Angew. Chem..

[28] Nicoludis J.M., Barrett S.P., Mergny J.L., Yatsunyk L.A. (2012). Interaction of human telomeric DNA with N-methyl mesoporphyrin IX. Nucleic Acids Res..

[29] Phatak P., Cookson J.C., Dai F., Smith V., Gartenhaus R.B., Stevens M.F., Burger A.M. (2007). Telomere uncapping by the G-quadruplex ligand RHPS4 inhibits clonogenic tumour cell growth in vitro and in vivo consistent with a cancer stem cell targeting mechanism. Br. J. Cancer.

[30] Murti K.G., Prescott D.M. (2002). Topological organization of DNA molecules in the macronucleus of hypotrichous ciliated protozoa. Chromosome Res..

[31] Prescott D.M. (1994). The DNA of ciliated protozoa. Microbiol. Rev..

[32] Paeschke K., Simonsson T., Postberg J., Rhodes D., Lipps H.J. (2005). Telomere end-binding proteins control the formation of G-quadruplex DNA structures in vivo. Nat. Struct. Mol. Biol..

[33] Schaffitzel C., Berger I., Postberg J., Hanes J., Lipps H.J., Plückthun A. (2001). In vitro generated antibodies specific for telomeric guanine-quadruplex DNA react with Stylonychia lemnae macronuclei. Proc. Natl Acad. Sci. U.S.A..

[34] Paeschke K., Juranek S., Rhodes D., Lipps H.J. (2008). Cell cycle-dependent regulation of telomere tethering in the nucleus. Chromosome Res..

[35] Fernando H., Rodriguez R., Balasubramanian S. (2008). Selective recognition of a DNA G-quadruplex by an engineered antibody. Biochemistry.

[36] Ladame S., Schouten J.A., Roldan J., Redman J.E., Neidle S., Balasubramanian S. (2006). Exploring the recognition of quadruplex DNA by an engineered Cys2-His2 zinc finger protein. Biochemistry.

[37] Biffi G., Tannahill D., McCafferty J., Balasubramanian S. (2013). Quantitative visualization of DNA G-quadruplex structures in human cells. Nature Chem..

[38] Henderson A., Wu Y., Huang Y.C., Chavez E.A., Platt J., Johnson F.B., Brosh R.M., Sen D., Lansdorp P.M. (2014). Detection of G-quadruplex DNA in mammalian cells. Nucleic Acids Res..

[39] Binz H.K., Stumpp M.T., Forrer P., Amstutz P., Plückthun A. (2003). Designing repeat proteins: well-expressed, soluble and stable proteins from combinatorial libraries of consensus ankyrin repeat proteins. J. Mol. Biol..

[40] Zahnd C., Amstutz P., Plückthun A. (2007). Ribosome display: selecting and evolving proteins in vitro that specifically bind to a target. Nat. Methods.

[41] Steiner D., Forrer P., Plückthun A. (2008). Efficient selection of DARPins with sub-nanomolar affinities using SRP phage display. J. Mol. Biol..

[42] Brooks T.A., Kendrick S., Hurley L. (2010). Making sense of G-quadruplex and i-motif functions in oncogene promoters. FEBS J..

[43] Wlodarczyk A., Grzybowski P., Patkowski A., Dobek A. (2005). Effect of ions on the polymorphism, effective charge, and stability of human telomeric DNA. Photon correlation spectroscopy and circular dichroism studies. J. Phys. Chem. B.

[44] Rheinnecker M., Hardt C., Ilag L.L., Kufer P., Gruber R., Hoess A., Lupas A., Rottenberger C., Plückthun A., Pack P. (1996). Multivalent antibody fragments with high functional affinity for a tumor-associated carbohydrate antigen. J. Immunol..

[45] Pack P., Müller K., Zahn R., Plückthun A. (1995). Tetravalent miniantibodies with high avidity assembling in Escherichia coli. J. Mol. Biol..

[46] Hammes G.G., Chang Y.C., Oas T.G. (2009). Conformational selection or induced fit: a flux description of reaction mechanism. Proc. Natl Acad. Sci. U. S. A..

[47] Catasti P., Chen X., Moyzis R.K., Bradbury E.M., Gupta G. (1996). Structure-function correlations of the insulin-linked polymorphic region. J. Mol. Biol..

[48] Ambrus A., Chen D., Dai J., Jones R.A., Yang D. (2005). Solution structure of the biologically relevant G-quadruplex element in the human c-MYC promoter. Implications for G-quadruplex stabilization. Biochemistry.

[49] Bah A., Azzalin C.M. (2012). The telomeric transcriptome: from fission yeast to mammals. Int. J. Biochem. Cell Biol..

[50] Maicher A., Lockhart A., Luke B. (2014). Breaking new ground: digging into TERRA function. Biochim. Biophys. Acta.

[51] Kummer L., Hsu C.W., Dagliyan O., Macnevin C., Kaufholz M., Zimmermann B., Dokholyan N.V., Hahn K.M., Plückthun A. (2013). Knowledge-based design of a biosensor to quantify localized ERK activation in living cells. Chem. Biol..

[52] Johnson J.E., Smith J.S., Kozak M.L., Johnson F.B. (2008). In vivo veritas: using yeast to probe the biological functions of G-quadruplexes. Biochimie.

[53] Wang Y., Patel D.J. (1993). Solution structure of the human telomeric repeat d[AG3(T2AG3)3] G-tetraplex. Structure.

[54] Parkinson G.N., Lee M.P., Neidle S. (2002). Crystal structure of parallel quadruplexes from human telomeric DNA. Nature.

[55] Phan A.T., Kuryavyi V., Luu K.N., Patel D.J. (2007). Structure of two intramolecular G-quadruplexes formed by natural human telomere sequences in K +solution. Nucleic Acids Res..

[56] Lim K.W., Amrane S., Bouaziz S., Xu W., Mu Y., Patel D.J., Luu K.N., Phan A.T. (2009). Structure of the human telomere in K+ solution: a stable basket-type G-quadruplex with only two G-tetrad layers. J. Am. Chem. Soc..

[57] Schonhoft J.D., Bajracharya R., Dhakal S., Yu Z., Mao H., Basu S. (2009). Direct experimental evidence for quadruplex-quadruplex interaction within the human ILPR. Nucleic Acids Res..

