# Supplementary material for: G-quadruplexes are specifically recognized and distinguished by selected designed ankyrin repeat proteins
Source: Nucleic Acids Res. 2014 Jul 22;42(14):9182–94. doi: 10.1093/nar/gku571 (PMC4132713; doi:10.1093/nar/gku571)
Supplement: SUPPLEMENTARY DATA [file supp_42_14_9182__index.html]

G-quadruplexes are specifically recognized and distinguished by selected designed ankyrin repeat proteins — G-quadruplexes are specifically recognized and distinguished by selected designed ankyrin repeat proteins — SUPPLEMENTARY DATA 

# G-quadruplexes are specifically recognized and distinguished by selected designed ankyrin repeat proteins

## SUPPLEMENTARY DATA

**Files in this Data Supplement:**

- SUPPLEMENTARY DATA
