# Supplementary material for: G-quadruplexes are specifically recognized and distinguished by selected designed ankyrin repeat proteins
Source: Nucleic Acids Res. 2014 Jul 22;42(14):9182–94. doi: 10.1093/nar/gku571 (PMC4132713; doi:10.1093/nar/gku571)
Supplement: SUPPLEMENTARY DATA [file supp_gku571_nar-00923-f-2014-File015.pdf]

Supplementary Figures and Tables

G-quadruplexes are specifically recognized and distinguished by selected designed ankyrin repeat proteins

Oliver Scholz, Simon Hansen, Andreas Plückthun*

Department of Biochemistry, University of Zurich, 8057 Zurich, Switzerland
* To whom correspondence should be addressed.

Email: plueckthun@bioc.uzh.ch

Table ST1. Selection conditions of those DARPins that were characterized in detail

	**Salt and target oligonucleotide**	**Library**
1C6	NaCl, tellong	N3C
1C7	NaCl, tellong	N3C
1C11	NaCl, tellong	N3C
1G2	KCl, tellong	N3C
1G11	KCl, tellong	N3C
1H4	KCl, teltt	N3C
2C10	NaCl, tellong	N3C
2D10	NaCl, teltt	N3C
2E4	KCl, tellong	N2C
2G7	KCl, tellong	N3C
2G10	KCl, tellong	N3C

Table ST2. Kinetic data obtained with SPR in TBS

tel

	k_{on} $M^{-1} \times s^{-1} \times 10^{6}$	k_{off} $s^{-1} \times 10^{-3}$	K_D $M \times 10^{-9}$
1C6	2.6 ± 0.9	40 ± 10	16 ± 2
1C7	1.2 ± 0.4	40 ± 1	37 ± 15
1C11	1.6 ± 1.1	74 ± 20	53 ± 22
1G2	1.0 ± 0.4	45 ± 21	53 ± 44
1G11	0.055 ± 0.0056	4.1 ± 2.9	72 ± 46
1H4	1.6 ± 1.0	82 ± 24	62 ± 34
2C10	1.5 ± 0.3	68 ± 10	48 ± 12
2D10	2.0 ± 0.4	64 ± 14	34 ± 16
2E4			
2G7	1.5 ± 0.1	30 ± 0	20 ± 2
2G10			

ILPR

	k_{on} $M^{-1} \times s^{-1} \times 10^{6}$	k_{off} $s^{-1} \times 10^{-3}$	K_D $M \times 10^{-9}$
1C6			
1C7	1.9 ± 0.6	62 ± 16	33 ± 8
1C11	1.3 ± 0.9	42 ± 2	43 ± 29
1G2	3.5 ± 0.7	90 ± 14	27 ± 9
1G11	0.14 ± 0.14	4.1 ± 1.8	44 ± 30
1H4			
2C10			
2D10			
2E4			
2G7			
2G10			

c-MYC

	k_{on} $M^{-1} \times s^{-1} \times 10^{-6}$	k_{off} $s^{-1} \times 10^{-3}$	K_D $M \times 10^{-9}$
1C6			
1C7	1.2 ± 0.6	79 ± 20	81 ± 38
1C11	2.1 ± 1.7	79 ± 27	49 ± 27
1G2	2.4 ± 0.9	95 ± 7	43 ± 19
1G11	0.12 ± 0.06	2.1 ± 0.9	19 ± 2
1H4			
2C10			
2D10			
2E4	2.7 ± 2.1	130 ± 30	64 ± 40
2G7			
2G10	0.95 ± 0.59	68 ± 2	90 ± 59

Table ST3. Kinetic data obtained with SPR in TBS-KCl

tel

	$k_{on,1}$ $M^{-1} \times s^{-1} \times 10^6$	$k_{off,1}$ $s^{-1} \times 10^{-3}$	$K_{D,1}$ $M \times 10^{-9}$	$k_{on,2}$ $M^{-1} \times s^{-1} \times 10^6$	$k_{off,2}$ $s^{-1} \times 10^{-3}$	$K_{D,2}$ $M \times 10^{-9}$
1C6						
1C7	1.5 ± 0.2	23 ± 1.2	15 ± 1	0.062 ± 0.035	0.28 ± 0.36	3.3 ± 3.0
1C11	2.6 ± 2.7	33 ± 38	11 ± 2	0.83 ± 1.27	14 ± 24	8.9 ± 8.3
1G2	3.6 ± 1.0	28 ± 7	7.9 ± 1.9	1.0 ± 0.2	3.3 ± 0.5	3.3 ± 1.1
1G11						
1H4	2.5 ± 0.8	36 ± 4	15 ± 4	0.11 ± 0.05	1.3 ± 0.1	14 ± 7
2C10	4.2 ± 1.0	83 ± 17	20 ± 1	0.24 ± 0.04	7.8 ± 5.1	35 ± 28
2D10	4.5 ± 1.0	78 ± 13	18 ± 3	0.29 ± 0.10	6.2 ± 2.4	22 ± 2
2E4	4.3 ± 5.7	6.4 ± 6.3	4.6 ± 4.7	3.7 ± 2.3	100 ± 83	42 ± 48
2G7	2.4 ± 1.1	22 ± 10	10 ± 5	0.55 ± 0.08	2.9 ± 0.6	5.5 ± 1.9
2G10	3.5 ± 2.7	141 ± 87	65 ± 69	0.21 ± 0.12	4.0 ± 4.7	15 ± 11

ILPR

	$k_{on,1}$ $M^{-1} \times s^{-1} \times 10^6$	$k_{off,1}$ $s^{-1} \times 10^{-3}$	$K_{D,1}$ $M \times 10^{-9}$	$k_{on,2}$ $M^{-1} \times s^{-1} \times 10^6$	$k_{off,2}$ $s^{-1} \times 10^{-3}$	$K_{D,2}$ $M \times 10^{-9}$
1C6						
1C7	2.4 ± 0.2	50 ± 3	21 ± 2	0.047 ± 0.011	0.61 ± 0.16	14 ± 8
1C11	9.3 ± 3.5	110 ± 50	12 ± 2	a)	a)	4.0 ± 4.6
1G2	16 ± 10	128 ± 25	9.4 ± 3.2	0.25 ± 0.21	0.68 ± 0.27	6.4 ± 7.4
1G11						
1H4						
2C10						
2D10						
2E4						
2G7						
2G10	8.5 ± 3.0	179 ± 36	22 ± 5	0.10 ± 0.08	1.1 ± 1.1	19 ± 24

a) low values indicate a different kinetic model of this DARPin

c-MYC

	$k_{on,1}$ $M^{-1} \times s^{-1} \times 10^6$	$k_{off,1}$ $s^{-1} \times 10^{-3}$	$K_{D,1}$ $M \times 10^{-9}$	$k_{on,2}$ $M^{-1} \times s^{-1} \times 10^6$	$k_{off,2}$ $s^{-1} \times 10^{-3}$	$K_{D,2}$ $M \times 10^{-9}$
1C6						
1C7						
1C11	3.7 ± 0.7	64 ± 21	17 ± 3	0.015 ± 0.026	0.10 ± 0.17	6.8 ± 4.3
1G2	6.6 ± 2.4	97 ± 17	16 ± 5	0.095 ± 0.039	1.2 ± 0.3	14 ± 4
1G11						
1H4						
2C10						
2D10						
2E4	2.7 ± 0.9	67 ± 10	28 ± 13	1.9 ± 3.2	4.2 ± 4.7	19 ± 27
2G7						
2G10	2.8 ± 2.1	97 ± 61	104 ± 150	0.66 ± 0.96	7.0 ± 9.3	15 ± 5

Figure Legends:

Figure S1-S6. k_{on}, k_{off} and K_D values calculated from SPR data. S1: TBS, *tel*; S2: TBS-KCl, *tel*; S3: TBS, *ILPR*; S4: TBS-KCl, *ILPR*; S5: TBS, *c-Myc*; S6: TBS-KCl, *c-Myc*. For measurements in TBS-KCl, a heterogeneous ligand model was used; values for both binding events are given. Order of a group of bars is always: k_{on}, k_{off}, K_D

Figure S7. Sequences of selected DARPins. The differences to the consensus at randomized positions (indicated by X in consensus) and framework mutations are shown. Residues that had been randomized in the original design are boxed. DARPins 2E4 contains two internal repeats (N2C), all others contain 3 internal repeats (N3C).

Figure S1 *tel*, TBS

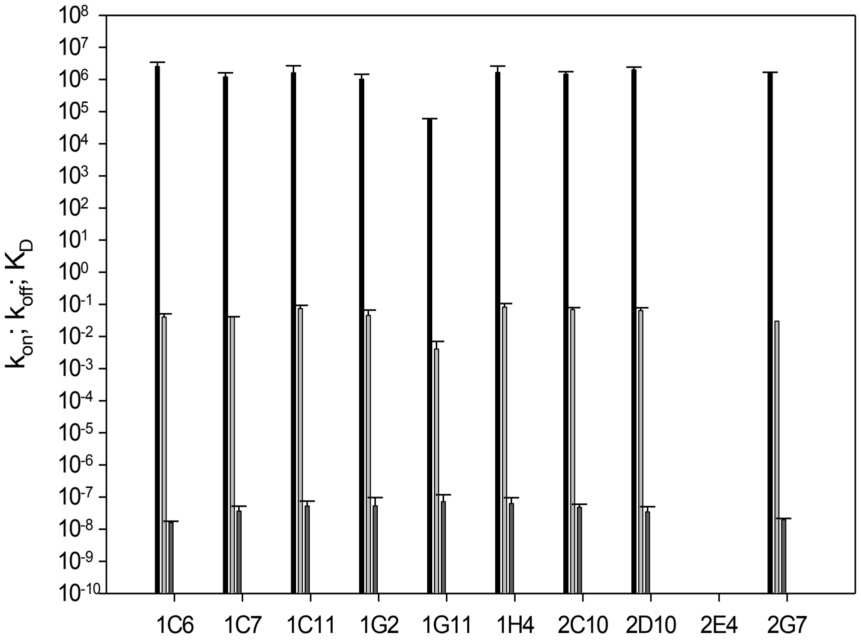

Figure S2 *tel*, TBS-KCl

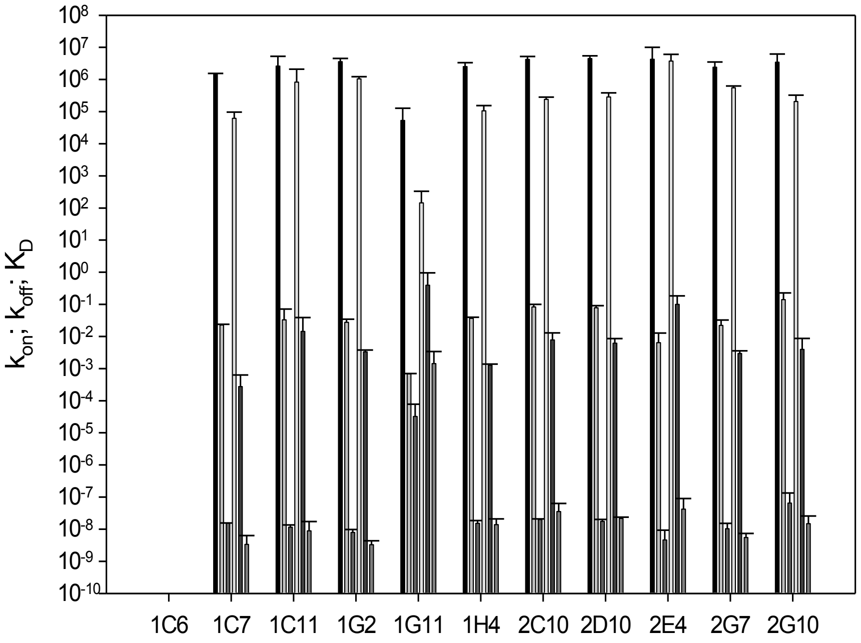

Figure S3 *ILPR*, TBS

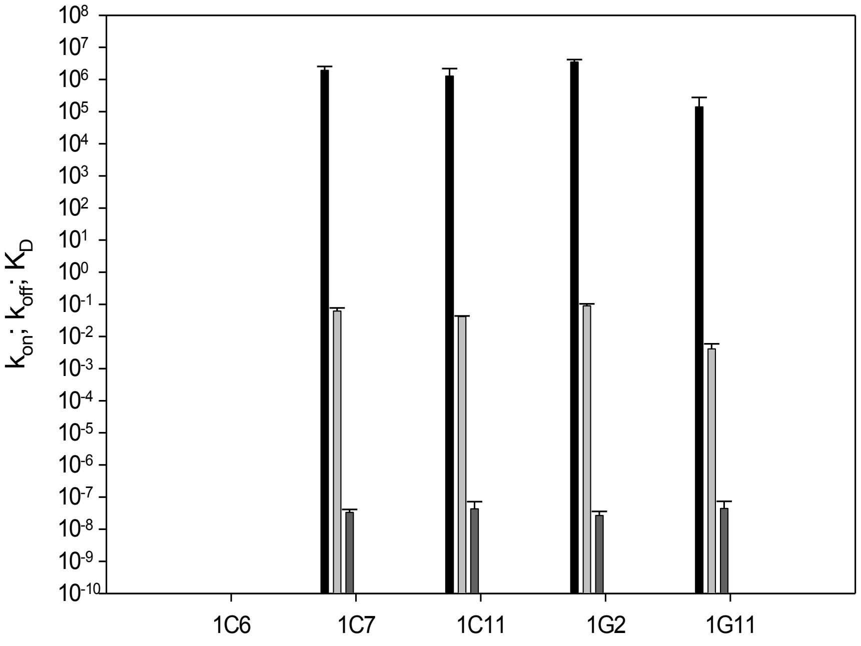

Figure S4 *IPLR*, TBS-KCl

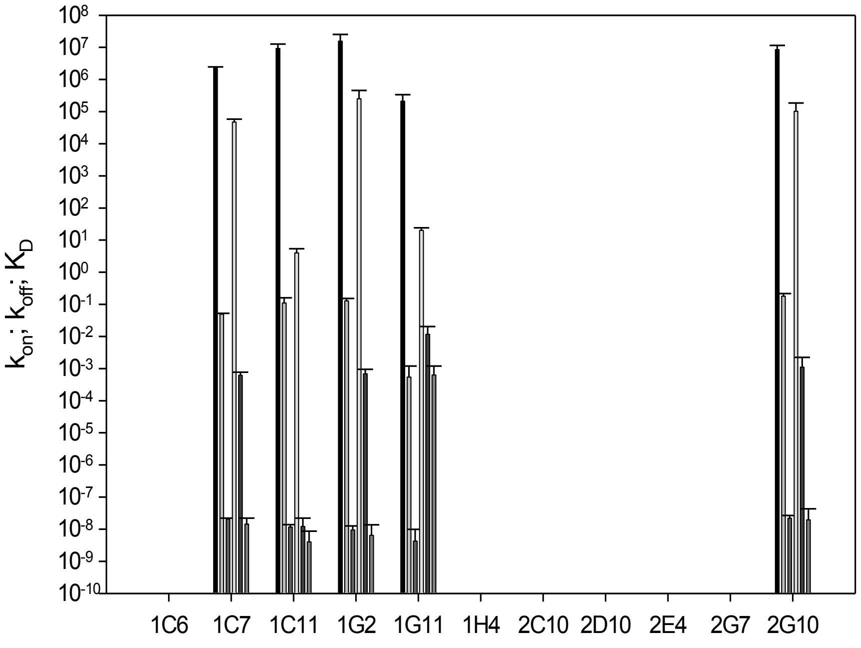

Figure S5 *c-Myc*, TBS

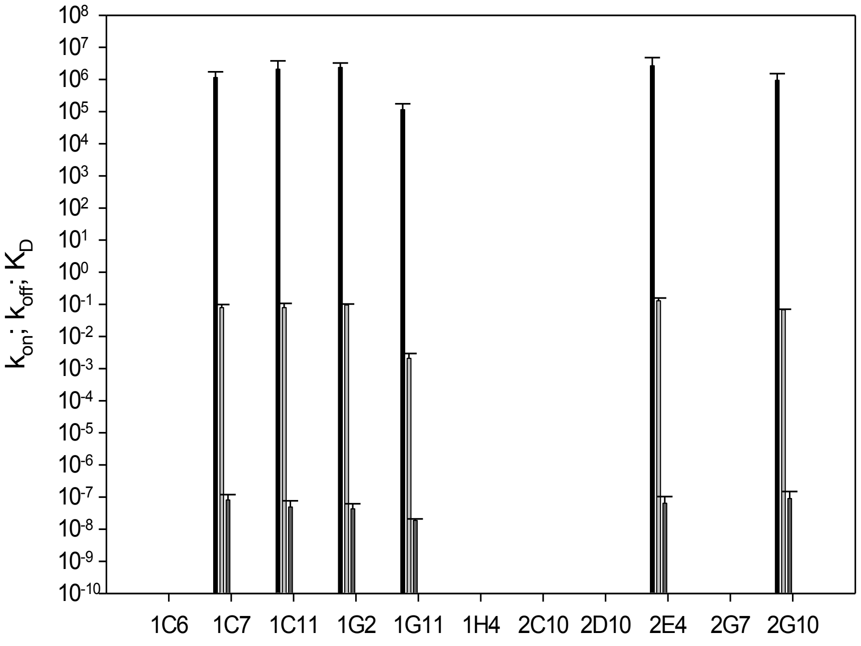

Figure S6 *c-Myc*, TBS-KCl

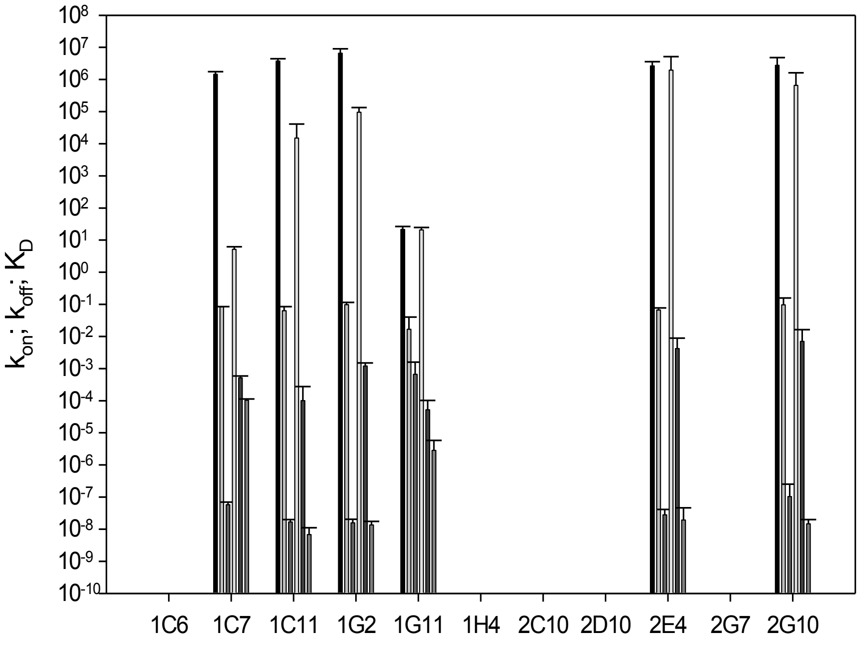

Figure S7

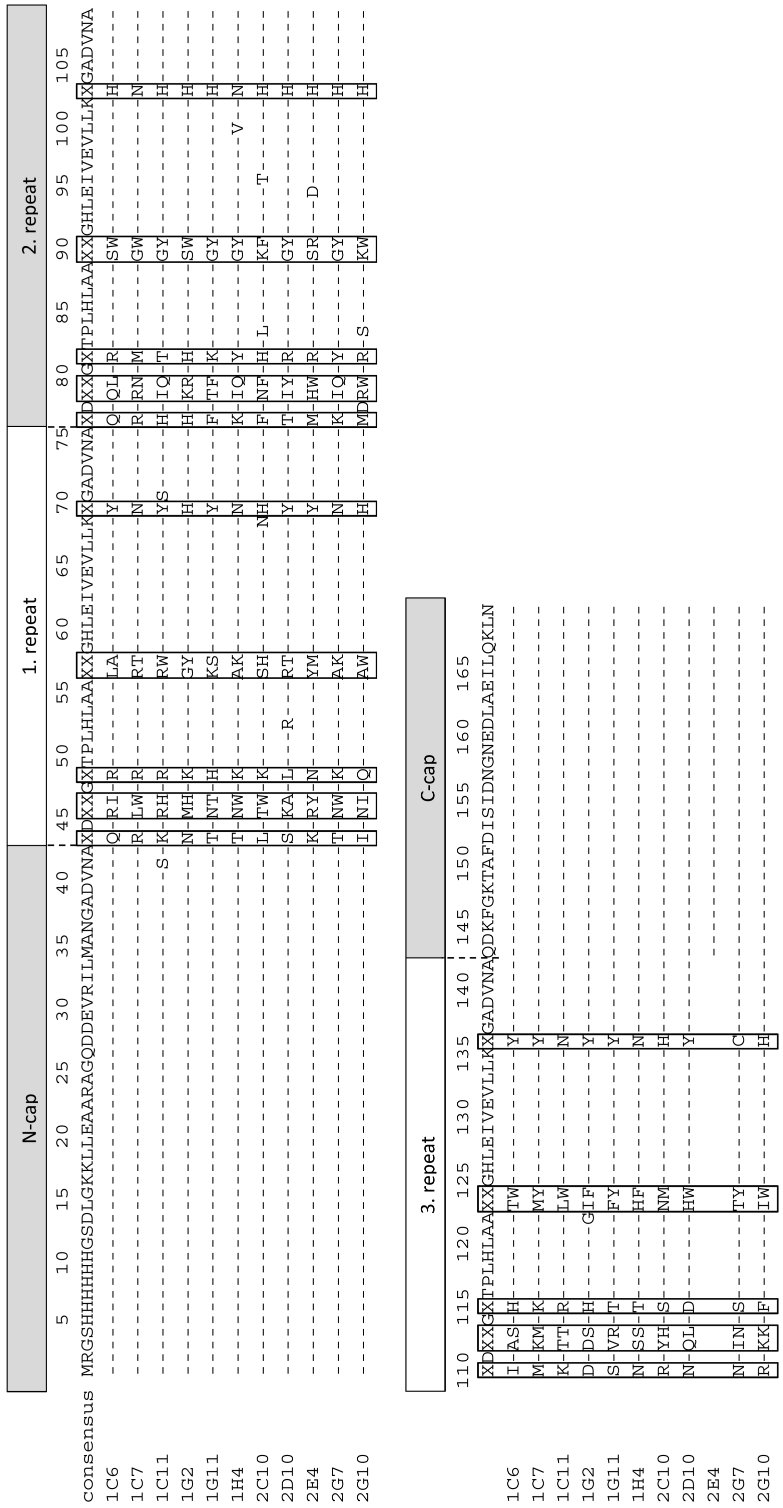
N-cap
1. repeat
2. repeat
3. repeat
C-cap
consensus
1C6
1C7
1C11
1G2
1G11
1H4
2C10
2D10
2E4
2G7
2G10
